# Magnetically Controllable Polymer Nanotubes from a Cyclized Crosslinker for Site-Specific Delivery of Doxorubicin

**DOI:** 10.1038/srep17478

**Published:** 2015-12-01

**Authors:** Ben Newland, Daniel Leupelt, Yu Zheng, Laurent S. V. Thomas, Carsten Werner, Martin Steinhart, Wenxin Wang

**Affiliations:** 1 Leibniz Institute of Polymer Research Dresden, Max Bergmann Centre for Biomaterials Dresden, Hohe Straße. 6, Dresden 01069, Germany; 2 Brain Repair Group, Schools of Biosciences, Cardiff University, Cardiff, UK; 3 Institut für Chemie neuer Materialien, Universität Osnabrück, Barbarastr. 7 D-49069, Osnabrück, Germany; 4 Melville Laboratory for Polymer Synthesis, Department of Chemistry, University of Cambridge, Lensfield Road, Cambridge CB2 1EW, UK; 5 The Charles Institute of Dermatology, School of Medicine and Medical Science, University College of Dublin, Dublin, Ireland

## Abstract

Externally controlled site specific drug delivery could potentially provide a means of reducing drug related side effects whilst maintaining, or perhaps increasing therapeutic efficiency. The aim of this work was to develop a nanoscale drug carrier, which could be loaded with an anti-cancer drug and be directed by an external magnetic field. Using a single, commercially available monomer and a simple one-pot reaction process, a polymer was synthesized and crosslinked within the pores of an anodized aluminum oxide template. These polymer nanotubes (PNT) could be functionalized with iron oxide nanoparticles for magnetic manipulation, without affecting the large internal pore, or inherent low toxicity. Using an external magnetic field the nanotubes could be regionally concentrated, leaving areas devoid of nanotubes. Lastly, doxorubicin could be loaded to the PNTs, causing increased toxicity towards neuroblastoma cells, rendering a platform technology now ready for adaptation with different nanoparticles, degradable pre-polymers, and various therapeutics.

In recent years, there has been a large research focus towards increasing the efficiency of current drugs and therapeutics. One method of improving efficiency is through site specific delivery of the drug, in order to reduce off-target effects^1^. Such targeted drug delivery would be particularly beneficial in anti-cancer applications where the side effects of a systemic action are problematic. Carbon nanotubes (CNTs) have been proposed as one such material for drug delivery^2^,^3^. Their small size and bulk production capabilities make them a convenient starting material for such biomedical applications, however, they hold the inherent drawbacks of toxicity^4^,^5^, functionalization requirement^6^, degradation issues^6^,^7^ and difficulty internalizing cargo^8^.

Polymer nanotubes (PNTs) could pose an attractive alternative through the ability to synthesize softer/weaker materials (where the high strength of CNTs is not required), circumvent the toxicity via the use of degradable polymer materials or have larger internal diameters for internal drug loading. Whilst PNT synthesis methods such as micropipette stretching techniques can yield hollow polymer nanomaterials^9^ a scalable method such as coating a template could be far more favorable for widespread application. Since the early uses of porous anodized aluminum oxide (AAO) as a template material^10^, much research has focused on template production of high aspect ratio materials using a variety of starting components^11^,^12^,^13^,^14^,^15^. Aside from polymer melt template wetting procedures which require high temperatures (depending on polymer type)^12^, polymerization within AAO pores usually requires modification of the template with a catalyst or initiator, prior to the reaction process^13^,^14^. In certain cases specific vinyl containing macromonomers have to be pre-synthesized in order for nanotubes to form^15^. Controlled living polymerization techniques such as atom transfer radical polymerization (ATRP) have also been used to synthesize polymer nanotubes but with either the formation of clumped aggregates of tubes (when an AAO template is used)^14^, or pristine tubes are formed which contain residual silica from the sacrificial template^16^ (a likely drawback for biomedical applications).

In this study we aimed to prove a concept that a simple polymerization technique could be used to form a photocrosslinkable ethylene glycol dimethacrylate (EGDMA) homopolymer which could be used to make PNTs. Furthermore, we aimed to functionalize the nanotubes with iron oxide nanoparticles for magnetic manipulation, and show that a large internal pore could be filled with an anticancer drug such as doxorubicin. We hypothesized that *in situ* deactivation enhanced ATRP (DE-ATRP) could produce a specific structure of poly(EGDMA) consisting of knots and branches allowing it to photocrosslink within AAO pores due to the high percentage of free vinyl groups in its structure. Our objectives were to: a) synthesize (and characterize) a homopolymer from a commercially available monomer in large quantities (typically >5 grams at a time), to prove the scalability of this approach, b) form (and characterize) pristine free standing (not aggregated) nanotubes using a single photocrosslinking step, c) demonstrate a simple *in situ* tube functionalization procedure and lastly, d) investigate whether these nanotubes could deliver doxorubicin to neuroblastoma *in vitro*.

## Results and Discussion

### Nanotube Synthesis and Characterization

**Figure 1:**
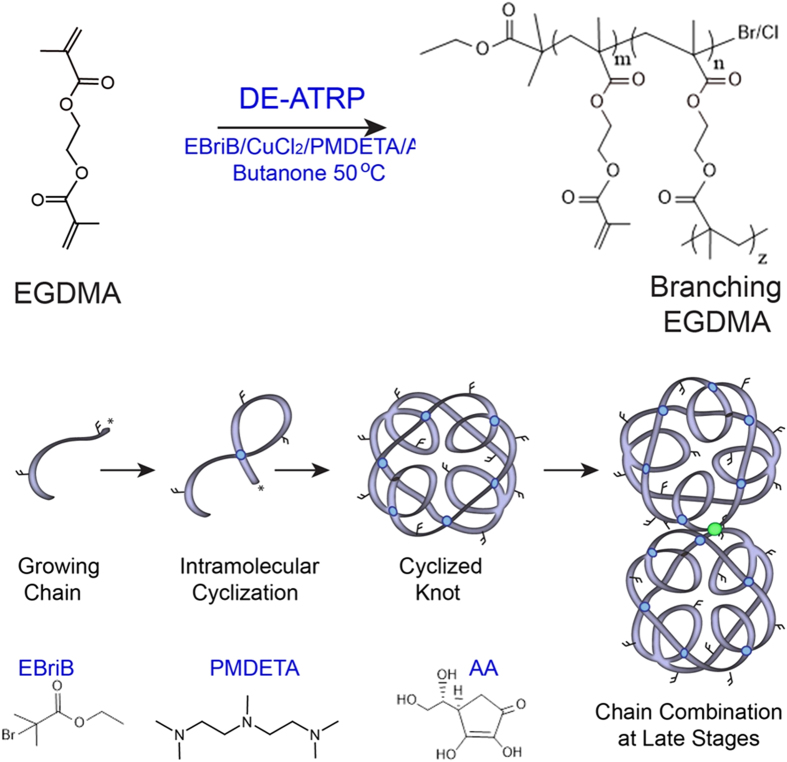
A simple “one-pot” route to a photocrosslinkable pre-polymer. The homopolymerization of EGDMA via *in situ* Deactivation Enhanced ATRP (DE-ATRP) allows the formation of a growing chain and intramolecular cyclizations. In the early reaction phases a knot structured polymer is produced, however, at later stages these knots combine via traditional intermolecular branches.

Much research into the delivery of therapeutics via nanotubes has focused on the use of graphitic carbon nanotubes with subsequent surface functionalization to covalently bind a cargo^17^. Carbon structures containing a nanoscopic open pore (termed carbon nanopipes) have been synthesized within an AAO template^18^,^19^,^20^ but this process requires carbon vapor deposition at high temperatures limiting the capacity for *in situ* functionalization of the tubes. Herein we develop a homopolymer designed to be photocrosslinkable within the pores of an AAO template and thus allow the formation of hollow nanotubes at room temperature. Figure 1 depicts the “deactivation enhanced” modification of atom transfer radical polymerization (ATRP) required for the synthesis of a homopolymer from the bifunctional vinyl monomer ethylene glycol dimethacrylate (EGDMA)^21^. Traditionally the inclusion of only a few percent of a multi-vinyl monomer in an ATRP co-polymerization would result in the formation of an insoluble crosslinked gel^22^ unsuitable for further study. However, recently we have shown that deactivation enhanced ATRP (DE-ATRP) can control the synthesis (reduce the kinetic chain length and therefore growth boundary) to such an extent as to allow homopolymerization of EGDMA into single soluble chains^21^,^23^. In this study, we synthesize these chains, which contain internal cyclizations (intramolecular crosslinks) and free (unreacted) vinyl groups depicted in Fig. 1 as overlapping chains (blue circles) and free double bonds sticking out of the chain. However, at later reaction stages, as free monomers become more exhausted and cyclized knot chain concentrations are high, the cyclized chains combine to form multiple knots joined by intermolecular crosslinks^24^, shown as a green circle in Fig. 1.

Gel permeation chromatography (GPC) analysis during the reaction process (Supplementary Information Fig. 1) clearly shows the transition from unimodal peaks of the single chain (1–3 hours), to the much broader peaks of the larger, polydisperse, multiple knot structures when chains combine (4–5 hours) (Supplementary Fig. 2). ^1^H NMR spectroscopy (Supplementary Fig. 3) shows the presence of unreacted vinyl groups within the polymer structure (78.7%) for subsequent photocrosslinking within the AAO template. The final polymer yield was 9.3 g (47% yield).

**Figure 2:**
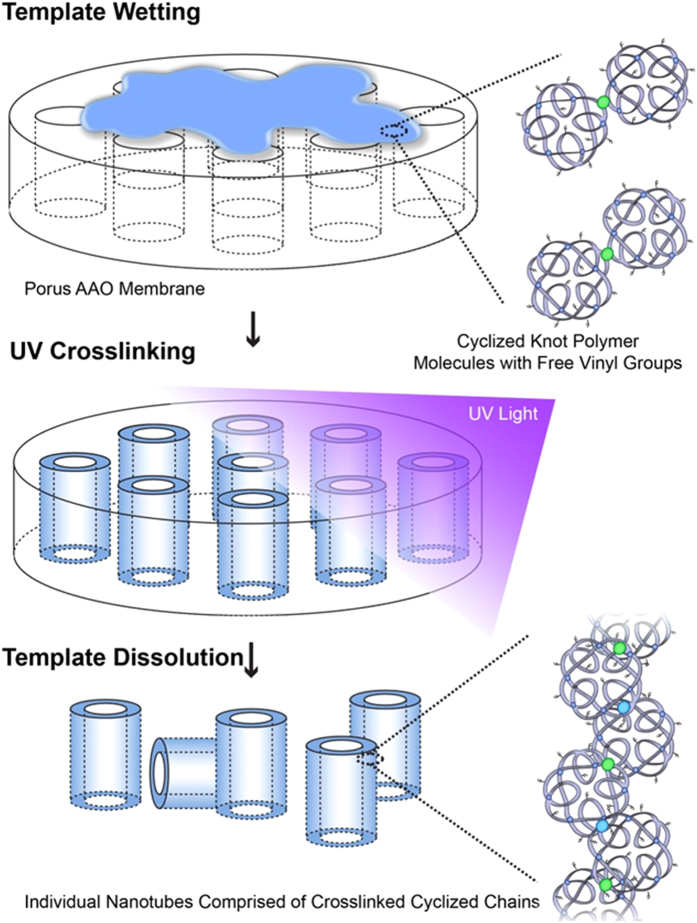
Schematic diagram depicting the facile route to polymer nanotube preparation, via photocrosslinking the knot polymers within AAO pores followed by template dissolution in sodium hydroxide.

**Figure 3:**
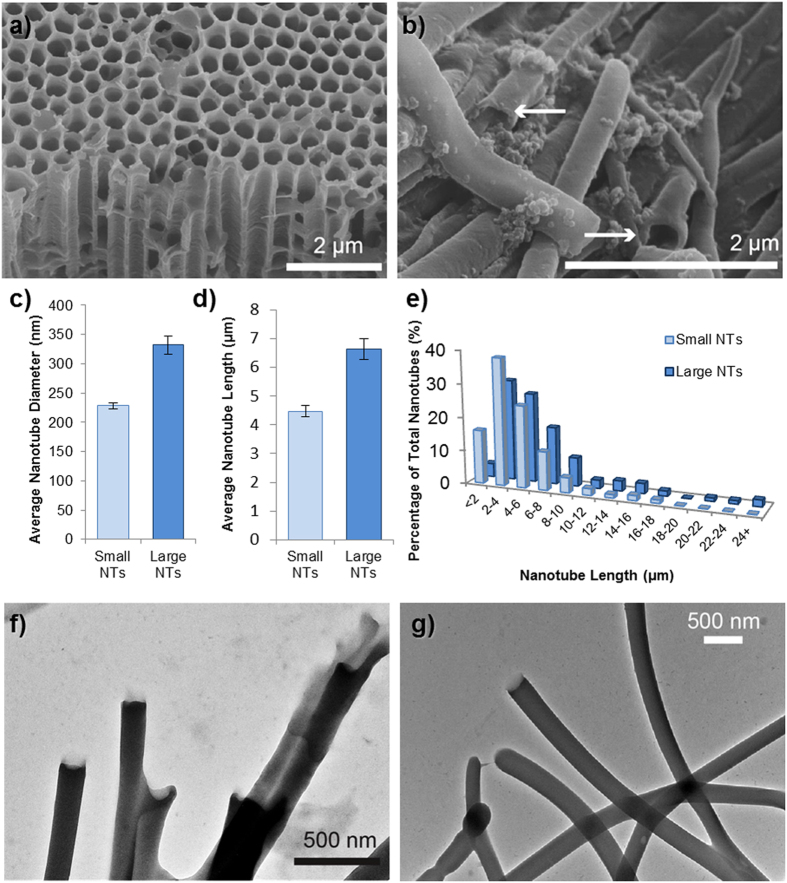
Polymer nanotube characterization. SEM images of (**a**) the 200 μm AAO template used to synthesize the “Small” polymer nanotubes and (**b**), the nanotubes after dissolution with arrows showing open tube ends. The diameter of the nanotubes could be adjusted depending on the template used (**c**) and the nanotubes with a larger diameter were also generally longer in length (**d**,**e**). TEM images (**f**) and (**g**) show “Small” and “Large” diameter nanotubes respectively, whilst (**g**) also exhibits some closed ends of the tubes.

Figure 2 shows the simple process used to synthesize the nanotubes, whereby the polymer is dissolved in a solvent, added to the template surface, and exposed to UV light. Figure 3a shows the “small” template, used to synthesize nanotubes of 200 nm diameter and Fig. 3b shows the nanotubes which have been released from the template by the addition of NaOH. Figures 3c and 1d show that the diameter and length of the nanotubes can be adjusted by varying the pore size of the AAO template used (length distributions shown in Fig. 3e). The “small” and “large” templates have pores suitable for the production of tubes with diameters of approximately 230 nm and 350 nm respectively as shown in Figs 3f and 1g respectively. Many tubes have a curved but regular morphology, with predominantly “open” ends, but with the occasional “test tube” like closed end as shown in Fig. 3g. One explanation for the formation of hollow tubes instead of solid nanowires is that the template is rapidly wetted by the pre-polymer mix in acetone (Supplementary Information Fig. 4), but the acetone evaporates quickly leaving the pre-polymer on the pore wall prior to crosslinking. Wall defects are rare (Supplementary Information Fig. 5) but may occur where the pore filling results in only a thin layer of polymer. Whilst explaining the difference in diameter between the “small” and “large” nanotubes is straightforward (i.e. it is dependent on the pore diameter of the template) the reason that the length distributions typically vary is less clear. Perhaps the mechanical stability of the smaller nanotubes is lower than that of the large nanotubes due to the greater confinement within the smaller pores suppressing the intermolecular entanglements or crosslinks. It was therefore desired to analyze if varying the pore depth of the template could alter the length distribution of the nanotubes. For this study, a standard deep pore “large” template (100 μm) was used to synthesize nanotubes which were compared with nanotubes produced in a shallow pore “large” template with a depth of 10 μm. This should, in theory, restrict the maximum nanotube length to 10 μm. Supplementary Information Fig. 6 shows that this restricted pore depth does indeed reduce the length distributions with a limited length dependent on the depth of the pore, but does not produce an exact predetermined nanotube length. The majority of tubes for either template are between 2–4 μm in length probably in part due to mechanical instability, in-complete template wetting (more layers could be applied in future), or breaking during template dissolution. However, since aspect ratio is likely to affect toxicity, this is a precise way to set a maximal length of the nanotubes.

### Effect of Nanotube Dispersions on Cell Viability

**Figure 4:**
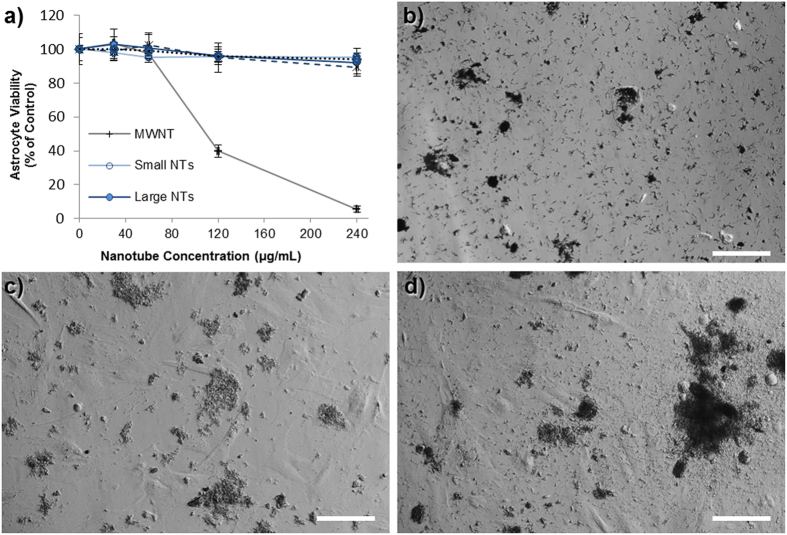
The polymer nanotubes show far lower cytotoxicity than MWNTs. The viability of primary astrocytes (a predominant cell type of the brain) extracted from the newborn rat midbrain is unaffected by the incubation with polymer nanotubes (**a**). Light microscopy analysis shows a heavy coverage of the nanotubes on the astrocytes ((**b**) = MWNT, (**c**) = “Small” polymer nanotubes, (**d**) = “Large” polymer nanotubes) at the highest concentration analyzed (240 μg/ml) (scale bars = 100 μm, n = 4).

The rationale for developing wide pore, polymer based nanotubes, is that they may be more suitable for drug delivery applications than carbon nanotubes, via enhanced drug loading and perhaps reduced toxicity. High risk neuroblastoma currently has poor prognosis following diagnosis so was chosen as the model system to investigate the use of these nanotubes as a drug delivery system. However, to investigate the cytotoxicity of the biomaterial itself (ie. nanotubes in their unloaded form), *in vitro* assessment was carried out using astrocytes: a non-cancerous cell type. The cytotoxicity of the nanotubes was measured using the PrestoBlue^®^ assay, after 24 hours of incubation of various concentrations of nanotubes on either primary cortical astrocytes (Fig. 4) or an astrocyte cell line (Supplementary Information Fig. 9) developed by the Fawcett lab^25^. These studies showed that both sizes of polymer nanotubes retained over 80% astrocyte viability up to a concentration of 240 μg/mL, whereas multi-walled carbon nanotubes (MWNT) of large diameter (170 nm by manufacturer’s definition) caused a dose dependent loss in viability. Brightfield images of the nanotube/cell incubations (Fig. 4b–d) show that 240 μg/mL is still a high enough concentration to result in a thick layer of nanotubes over the cell and well surface, with large aggregations also visible. The nanotubes themselves disperse well in solution (Supplementary Information Fig. 8) as measured by DLS and via visual observations over time. Since poly(ethylene glycol) is often used to functionalize materials for reduced aggregation, the fact that these tubes are predominantly ethylene glycol based may explain this good dispersion. However, the dispersion is concentration dependent as high concentrations such as 4 mg/ml will aggregate throughout the day. The difference in cytotoxicity caused by the polymer nanotubes compared to the MWNTs may be due to a number of factors, with the obvious starting point of differing material compositions (EGDMA vs graphitic carbon). In addition, the aspect ratio is much smaller for the polymer nanotubes, which is likely to explain the reduced toxicity. However, during transmission electron microscopy (TEM) analysis an interesting observation made showing the flexible nature of the polymer nanotubes. Where an area of the carbon film contained a tear the nanotubes either followed the contour of the curling film, or, if they spanned the film tear, they would stretch as the electron beam caused a widening of the tear (Supplementary Information Fig. 10). This purely qualitative analysis may however give an insight into the reduced toxicity. One could speculatively say that the combination of low aspect ratio and nanotube flexibility renders the material less toxic to cells at the concentrations analyzed.

### Synthesis of Magnetically Controllable Nanotubes

**Figure 5:**
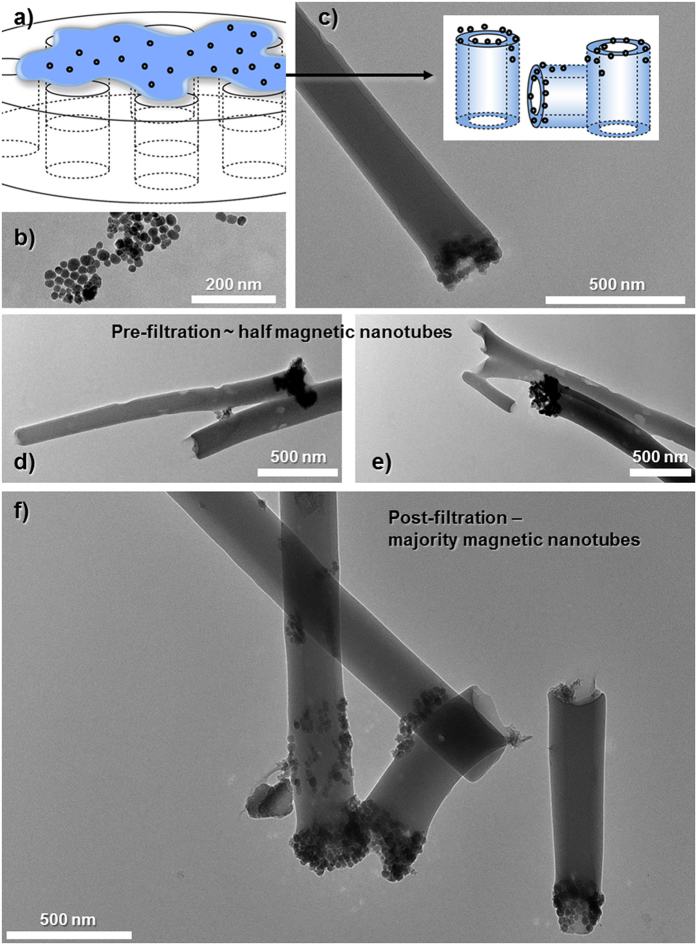
Schematic depiction ((a,c) insert) of functionalizing the nanotubes with magnetite. TEM images of: (**b**) magnetite nanoparticles, (**c**) a functionalized nanotube with a magnetic end, (**d**,**e**) sample images of magnetic nanotubes before magnetic filter separation and, (**f**) magnetically functionalized nanotubes after purification.

The process of filling the nanotubes with ferric oxide (Fe_2_O_3_) is depicted in Fig. 5a, where Fe_2_O_3_ nanoparticles are added into the polymer solution prior to crosslinking. A previous study into the formation of microscale magnetic manipulators used electrospinning to form polymer fibers containing superparamagnetic cobalt nanoparticles^26^. Although they demonstrated the connection of hippocampal neurons, the materials produced were micron scale, not hollow, and had to be cut into sections with a razor. Here we use an *in situ* filling mechanism that allows tube formation and magnetic functionalization. TEM analysis of the nanoparticles alone (Fig. 5b) shows a range of sizes but all are far below the 200 nm pores of the smallest AAO template used. Nanotubes could be produced which contained Fe_2_O_3_ at one end as shown in Fig. 5c–e. However, approximately half of the nanotubes appeared not to be functionalized as no nanoparticles could be observed at the tube ends. To sort the magnetic nanotubes from the non-magnetic nanotubes, a magnetic cell sorter was used (Supplementary Information Fig. 11). In this way, nanotubes containing Fe_2_O_3_ were attracted to the filter (under the effect of an external magnetic field) whilst the non-functionalized nanotubes passed through. After removal of the external magnetic field the functionalized nanotubes could be passed into a separate Eppendorf tube. 46% of the nanotubes were functionalized (Supplementary Information Fig. 11) and collected for subsequent analysis with Fig. 5f showing three such tubes together. Since none of the TEM images of the functionalized tubes either before or after filtration sorting showed free nanoparticles it can be assumed that all were incorporated during the photo-crosslinking. Thus an approximation of the weight percentage of Fe_2_O_3_ per nanotubes can be calculated from the average weight of the functionalized nanotube pellet. The value of 2.7 wt% Fe_2_O_3_ incorporation can only be taken as an approximation across the average of six batches of functionalized nanotubes. Since only about a half of the nanotubes was functionalized and only at one end, it would suggest that infiltration of the Fe_2_O_3_ nanoparticles into the template pores is poor and would have to be further improved if a greater weight percentage of Fe_2_O_3_ was desired.

### Doxorubicin Loading and Magnetic Focusing of the Nanotubes

Patients with cancers such as glioblastoma multiforme typically suffer extremely poor prognosis, therefore more effective therapies are being sought^27^. Research fields such as nanomedicine seek to improve the delivery efficacy of anti-cancer therapeutics by the development of nanoscale drug carriers^28^. Herein we analyze whether the polymer nanotubes can be loaded with a model anticancer drug doxorubicin (preferred for its intrinsic fluorescence allowing easy detection of the loaded nanotubes) and whether *in vitro* delivery can be achieved to model human cancer cells SH-SY5Y neuroblastoma.

**Figure 6:**
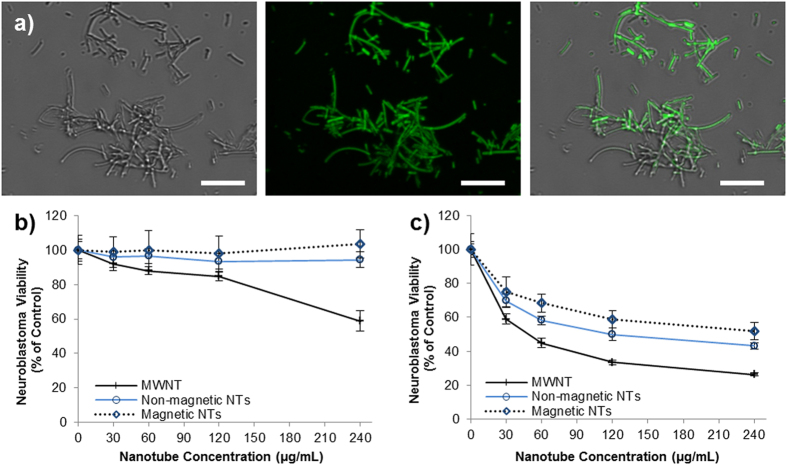
Nanotubes can be loaded with doxorubicin to mediate neuroblastoma cell death. High power (100× lens and 4× optical zoom) brightfield ((**a**) left) and fluorescent ((**a**) middle) microscopy of doxorubicin loaded nanotubes, visible via the intrinsic fluorescence of doxorubicin (emission at 488 nm: overlay (**a**) right). Both magnetic and non-magnetic nanotubes show no cytotoxicity towards SHSY5Y neuroblastoma at all concentrations tested (**b**), but cause over 40% loss in viability when incubated for 24 hours with doxorubicin prior to cell treatment (**c**) (n = 4, scale bars = 5 μm).

Dried nanotubes could be filled with a solution of doxorubicin in water by simple re-dispersion in the solution. For cytotoxicity experiments a high concentration of doxorubicin loading solution was chosen (1.5 mg/mL), however, for specific loading and release analysis a much lower concentration of 80 μg/mL was used to allow accurate detection of how much doxorubicin left the loading solution to enter the nanotubes. After loading the nanotubes with the doxorubicin solution, the nanotubes were centrifuged and washed with PBS extensively to remove any free doxorubicin. Fluorescent microscopy imaging (Fig. 6a) showed the doxorubicin loaded nanotubes to have a fluorescent emission at 488 (FITC filter) due to the intrinsic fluorescence of doxorubicin, but one cannot distinguish if the doxorubicin is within the pore or loaded to the tube wall. Doxorubicin uptake by the nanotubes, could be quantified by the change in absorbance of a doxorubicin loading solution at a concentration of 80 μg/ml, before and after the addition of empty nanotubes. An average of 38 ng of doxorubicin was loaded per μg of nanotubes (see Supplementary Table 4), and the release of the drug could be observed over a period of 8 days (maximum time analyzed) (Supplementary Fig. 12). Although it is unclear what drives such efficient loading (see Supplementary Fig. 12 insert to see how dark red the nanotube pellet becomes), others have reasoned that loading to polymers occurs via hydrophobic interaction between the doxorubicin and the polymer^29^. It is interesting to note that the polymers in that study^29^ were of similar compositions to the EGDMA nanotubes in terms of ethylene glycol was the main component, and poly(propylene glycol) was used to adjust the hydrophobicity (EGDMA is more hydrophobic than PEG). The sustained release profile observed may suggest that the doxorubicin is up-taken within the pore (not just surface loading) so that the 1D effect of a single or double open tube end (i.e. low pore surface to internal volume ratio - slow release) prolongs the release, as oppose to absorption to the nanotube wall (high surface area to volume ratio – quick release).

Doxorubicin loaded nanotubes (both magnetic and non-functionalized) showed anti-cancer activity against the human neuroblastoma derived cell line (SH-SY5Y cells, Fig. 6c). Additionally, the MWNT toxicity appeared enhanced after exposure to doxorubicin indicating that some doxorubicin may be present in the media, or loaded to the MWNT despite three washes (no fluorescence could be detected for the MWNT). At a nanotube concentration of 240 μg/mL neuroblastoma viability could be reduced to 52% (magnetic nanotubes) or 43% (non-functionalized nanotubes), compared to untreated control cells. As Fig. 6b shows, this anti-cancer activity is not a function of the toxicity of the nanotubes themselves, which show no significant toxicity towards the neuroblastoma even at 240 μg/mL. For comparison, free doxorubicin was incubated with SHSY-5Y cells at a variety of concentrations (see Supplementary Fig. 16) showing that the 42% and 52% viability is equivalent to approximately 0.4–0.5 μg/mL of free doxorubicin.

Another research field which has, of late, received much attention is that of targeted therapies. Designing targeted drug delivery systems can involve the targeting of the delivery vector to the target organ (for instance crossing the blood brain barrier to the brain), or to the specific cell, or a combination of both. This study aimed to make nanotubes that could be controlled/targeted via an external magnetic field. However, in principle the polymerization strategy could allow further modification of the polymer surface with antibody fragments or cell penetrating peptides via the free vinyl groups within the polymer structure (Supplementary Information Fig. 7 and Supplementary Table 3). The inclusion of iron oxide nanoparticles in material constructs can allow visualization of the carrier^28^, or magnetic manipulation^18^ or controlled drug release^30^. A recent study showed that ferrofluid (Fe_3_O_4_) could be added to carbon nanotubes formed by chemical vapor deposition within an AAO template^18^ (previously termed carbon nanopipes^19^,^20^). Herein by contrast, iron oxide nanoparticles were used to functionalize the nanotube walls via an *in situ* method without filling the pore space required for drug loading. It should be noted here, that although by the method described herein ~50% of the nanotubes were functionalized with the iron oxide nanoparticles, it is unclear to the authors how a greater filling can be achieved. Increasing the concentration of the nanoparticles, or adding the nanoparticles directly to the polymer solution prior to crosslinking showed no increase in loading efficiency. However, the method of magnetically sorting the nanotubes allowed subsequent experiments to be performed with pure solutions of functionalized nanotubes.

**Figure 7:**
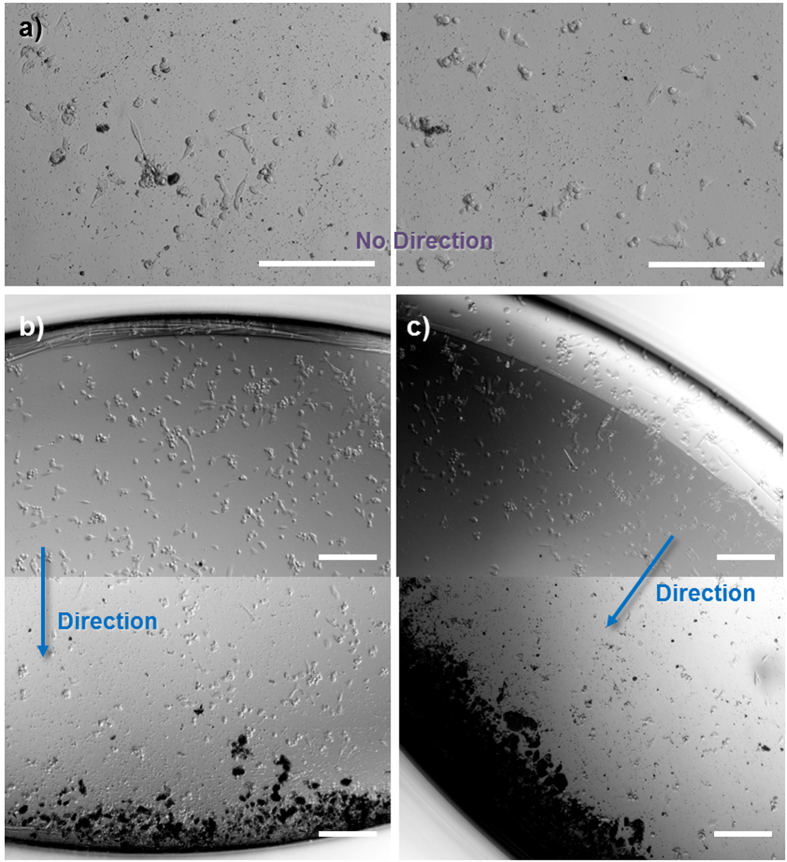
Magnetically controlled nanotubes can be directed within a well plate. Light microscope images of doxorubicin loaded magnetically functionalized nanotube clusters, which, in the absence of an external magnetic field spread evenly across a well plate (**a**). In contrast, doxorubicin loaded magnetic nanotubes can be guided away from one side of the well (upper image of (**b**)) towards the opposite side of the same well (lower image of (**b**). This could be repeated in different directions ((**c**) and SI Figure S14) depending on the placement of the external magnetic field (example images from 6 replicates, scale bars = 200 μm).

**Figure 8:**
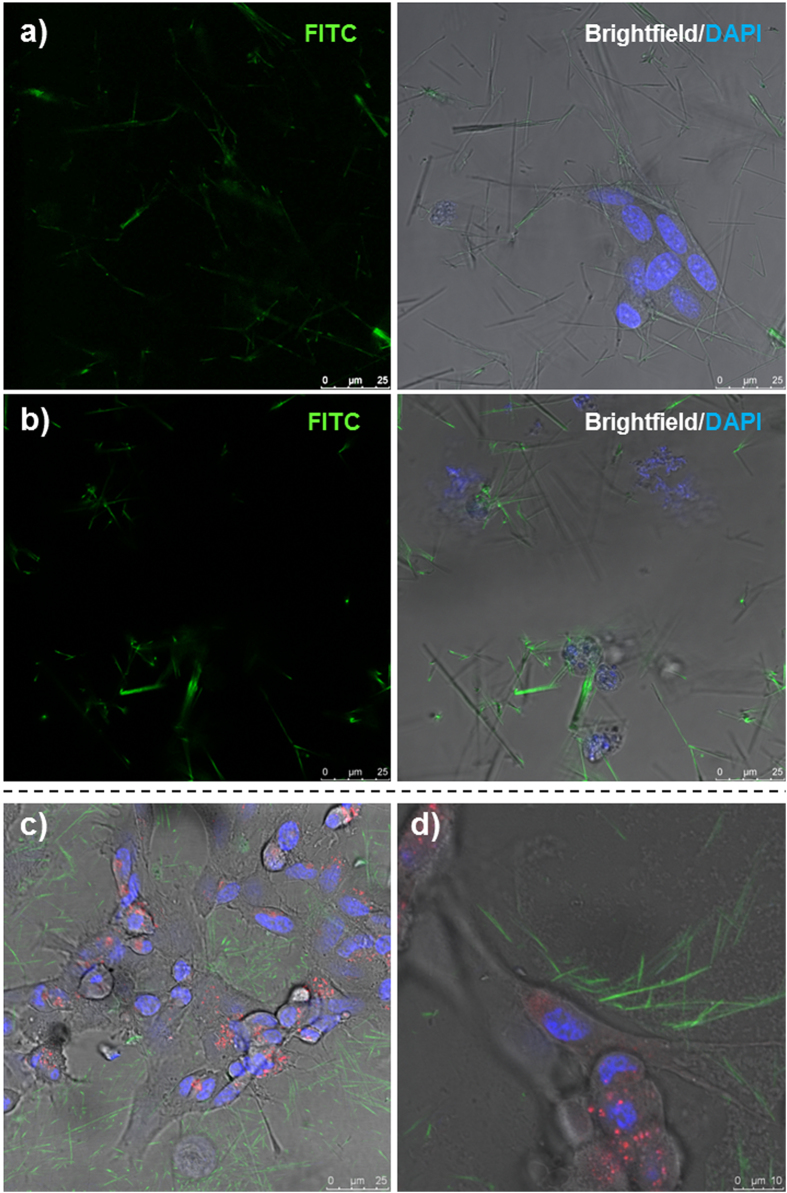
Doxorubicin loaded magnetic nanotubes achieve cell death without the uptake of the nanotubes themselves. Light and fluorescent microscopy images of SHSY-5Y cells incubated for 2 hours (**a**) or 24 hours (**b**) with doxorubicin loaded nanotubes at a concentration of 240 μg/mL (**b**). The intrinsic fluorescence of doxorubicin could be observed in the nanotubes at a wavelength of 488 nm and overlaid with bright field images merged with DAPI nuclear stain (right hand images) to show that many cells are affected by the doxorubicin loaded nanotubes after a 24 hour incubation period. A separate experiment was performed (**c**) with fluorescein labeled nanotubes (green) containing no doxorubicin, where cells were labelled with the DAPI nuclear stain and the lysosome tracker (LysoTracker® Deep Red), showing that despite nanotubes residing in the vicinity of the cells, no cell uptake of the nanotubes can be observed.

Non-functionalized nanotubes typically show an even spread across the cell monolayer (Supplementary Information Fig. 13). Functionalized nanotubes also show this even spread in the absence of an external magnetic field (Fig. 7a); however, the placement of the well plate upon a magnetic stirrer begins to drive the nanotubes in the direction determined by the magnetic field (see Supplementary Information Video 1). The nanotubes collect over at one side of the well plate (Fig. 7b,c, and Supplementary Information Fig. 14), which, despite an initial even covering, results in the opposite side of the well being completely devoid of nanotubes. These experiments highlight the ability of these nanotubes to be targeted to specific regions of cells *in vitro*, showing that the toxic effects of the doxorubicin can be localized instead of spread throughout the well. Finally, we show that doxorubicin released from non-functionalized and functionalized doxorubicin loaded nanotubes can be up-taken by the neuroblastoma cells by fluorescent microscopy without the up-take of the nanotubes themselves (Fig. 8 and Supplementary Information Fig. 15). At the highest concentration (240 μg/mL) most cells were shown to have up-taken doxorubicin as green fluorescence can be seen co-localizing with the cell regions shown by the brightfield images. However, at this concentration, empty nanotubes fluorescently labelled with fluorescein could be observed in the vicinity of the cells, but not within the lysosomes (indicated by LysoTracker® Deep Red, Life Technologies). This important finding indicates that the polymer nanotubes can act as a nanoscale carrier to deliver doxorubicin to cells without cell uptake or associated complications.

In conclusion a simple modification of atom transfer radical polymerization is used to make a photocrosslinkable polymer. A solution of this polymer can be crosslinked within the pores of an AAO template either in the presence or absence of iron oxide nanoparticles. The polymer nanotubes tubes released by dissolving the AAO template can be filled with doxorubicin, can cause dose dependent toxicity (otherwise absent if doxorubicin is absent) and can be magnetically directed to one side of the well plate to target doxorubicin delivery *in vitro*. This simple platform technology allows a wide range of further investigations such as functionalization with super paramagnetic iron oxide nanoparticles, or gold nanorods for combined hyperthermia and drug delivery applications.

## Methods

### Materials

Ethylene glycol dimethacrylate (EGDMA) (Aldrich) was used as the monomer, and ethyl 2-bromoisobutyrate (EBriB, 98%, Aldrich) was used as the initiator. The ligand pentamethyldiethylenetriamine (PMDETA, 99%, Aldrich), the catalyst copper(II) chloride (CuCl_2_, 97%, Aldrich), the reducing agent L-ascorbic acid (AA, 99%, Aldrich), and solvent 2-Butanone (HPLC grade, Aldrich), were all used as received. For polymer purification and analysis *n*-hexane (ACS reagent grade, Aldrich), dichloromethane (ACS reagent grade, Aldrich) and *d*-Chloroform (99.8%, Aldrich) were used as received. For nanotube synthesis either self-ordered AAO templates prepared according to procedures reported previously^31^,^32^ or Whatman™ Anodisc™ filter membranes (0.2 μm pore, Fischer Scientific) were used.

### Polymer Synthesis

Homopolymerization of EGDMA was carried out via the deactivation enhanced ATRP strategy outlined previously^21^. The initiator (ethyl 2-bromoisobutyrate (EBriB)) to monomer (ethylene glycol dimethacrylate (EGDMA)) ratio was 1:50. Table 1 in the Supplementary Information shows the ratio of all the reaction components along with the corresponding mass used. Firstly EGDMA (100 mmol) was weighed out into a clean two neck round bottomed flask, then the ligand (pentamethyldiethylenetriamine (PMDETA) 0.25 mmol) was added along with the catalyst (copper chloride (CuCl_2_, 0.25 mmol). EBriB was then added (2 mmol) along with the solvent butanone (50 mL) and the flask was de-gassed by bubbling nitrogen gently through the liquid. After 20 minutes the reaction was started by adding the reducing agent ascorbic acid (0.05 mmol) in a 100 mg/ml water solution carefully into the flask. Small samples (0.5 mL) were removed every hour to monitor the reaction progress by gel permeation chromatography (GPC) and the reaction was allowed to continue until very broad peaks were observed (multi-knot polymer^24^) (Supplementary Information Fig. 1). The experiment was stopped by opening the flask and exposing the catalyst to air. This was then diluted with butanone and passed through a column of alumina for chromatography and dripped into a large excess of hexane to remove the free monomer. The precipitated polymer was then filtered through filter paper under vacuum and allowed to dry. The yield was obtained by gravimetric analysis.

### Determination of Molecular Weight by GPC

The small samples taken from the reaction vessel were diluted in DMF and filtered through aluminia for chromatography before analysis. The molecular weight and molecular weight distribution of each sample was determined using a Varian 920-LC instrument with a refractive index detector (RI) calibrated with linear polystyrene standards. Chromatograms were run at 50 °C using DMF as eluent with a flow rate of 1 ml/min.

### Nuclear magnetic resonance (NMR) spectroscopy

The polymer composition was assessed by ^1^H NMR spectroscopy, by dissolving the purified polymer in deuterated chloroform and reporting all chemical shifts in ppm relative to TMS. SI Equation 1 was used to determine the percentage of polymer composition.

### Synthesis of Polymer Nanotubes

5 μL of poly(EGDMA) (5% w/v in acetone) was added to the top of an AAO template and spread carefully with the pipette tip. The template was then flushed with nitrogen and exposed to UV light for 10 minutes (Linos, Delolux 04, λ = 315–500 nm, intensity 8000 mW/cm^2^) to crosslink the polymer within the template pores. To obtain free standing nanotubes the AAO template was submerged in 1.5 mL of NaOH (1 M) for 15 minutes and briefly sonicated in a sonication bath without any prior template polishing. Purification of the nanotubes was performed by transferring the nanotube/NaOH mix to an Eppendorf tube and centrifuging for 10 minutes at 13,000 rpm. The supernatant was removed and the replaced with water, then the process was repeated twice with ethanol. To obtain a dry weight for subsequent study, the Eppendorf was first weighted then re-weighed after the ethanol had evaporated in under laminar flow. Fluorescently labelled nanotubes for cell up-take analysis were performed as above but with the inclusion of 2.5 wt% of fluorescein co-acrylate (Sigma) prior to UV crosslinking within the template.

### Synthesis of Magnetically Controllable Nanotubes

5 μl of 1.5 mg/mL magnetite (Fe_2_O_3_ nanopowder – Sigma) in DMF was first added to the AAO template and subjected to sonication, then 5 μL of poly(EGDMA) (10% w/v in acetone) was added on top. The magnetic nanotubes were removed from the template and purified as outlined above. To separate magnetic nanotubes from non-magnetic nanotubes, the MACS miltenyi biotec equipment was used (see Supplementary Information Fig. 6) which is normally used to magnetically sort cells. The column was first washed with deionized water, then 1 mL of nanotube suspension was added and passed through the column. Non-magnetic nanotubes were collected underneath in an Eppendorf tube, and one water wash was performed to ensure all non-magnetic nanotubes had passed through. Then the column was removed from the magnet and a further 1 mL of water was added and pushed through into a separate Eppendorf to flush out the magnetic nanotubes. These were then washed twice with ethanol and allowed to dry as described above.

### Characterization of Nanotubes

Samples were prepared for scanning electron microscopy (SEM) and transmission electron microscopy (TEM) by dropping 10 μl or 5 μl of a nanotube suspension (in ethanol) onto an SEM stub or carbon TEM grid respectively. Once fully dry, the sample for SEM was gold coated (SCD 050, BAL-TEC) for 60 seconds, and imaged using a Philips Environmental SEM (XL30) but in SEM mode. TEM images were obtained using a Zeiss Libra 200 TEM with an accelerating voltage of 200 kV using the contrast aperture when necessary. Length and diameter measurements were made using ImageJ software. Five dynamic light scattering (DLS) experiments were performed using a DynaPro NanoStar photospectrometer (WYATT Technology Corporation, USA), with nanotubes at a concentration of 1 mg/ml in PBS.

Raman spectroscopy analysis of the pre-polymer and nanotubes was performed using the Confocal Raman Microscope alpha 300 R (WITec GmbH, Ulm, Germany) equipped with a laser with an excitation wavelength of 785 nm and a laser power of 500 μW. Samples were measured with a 20× objective and an integration time of 0.5 s for a single scan in the wavelength region from 200 to 1800 cm^−1^. For each spectrum 200 accumulations were performed.

### Cell Culture

Primary cortical astrocytes were extracted from newborn rat pups (day 3) by a combination of two protocols^33^,^34^ as reported previously^35^. Both primary astrocytes and SHSY-5Y (Sigma) neuroblastoma cells were cultured in 50% Dulbecco’s Modified Eagles Medium (DMEM) as a 1:1 mix with F12 Ham (Gibco), 10% filtered fetal bovine serum (FBS) (Gibco) and 1% Penicillin/Streptomycin (P/S) (Gibco) at 37 °C with 5% CO_2_ using standard cell culturing techniques.

### Doxorubicin Loading and Release

Doxorubicin (LC Laboratories) was dissolved in deionized water to a final concentration of 1.5 mg/mL. The dried pellet of nanotubes were simply re-suspended in the doxorubicin solution and left at room temperature overnight. The tubes were then washed three times by centrifugation (13,000 rpm), supernatant removal, and re-suspension in sterile PBS. After the final wash, the nanotubes were re-suspended in the cell culture media described above. Loaded nanotubes were visualized using a confocal laser scanning microscope in both brightfield mode and with the FITC filter, using a 100× lens and 4× digital zoom. Unloaded nanotubes were used as a comparison (data not shown) to check for the possibility of nanotube auto fluorescence (none observed). For specific loading and release analysis, a lower concentration of doxorubicin (80 μg/ml) was used to calculate the percentage of the loading solution entered the nanotubes without absorbance maxima being reached. 263 μg of the nanotubes were incubated in 200 μl of doxorubicin and incubated at 37 °C for 48 hours, before being removed by centrifugation and washed. The absorbance of the loading solution, was measured before and after incubation to determine the amount loaded to the nanotubes. The nanotubes were then washed in PBS, before fresh PBS was added for a release study over a period of one week (at 37 °C), with samples being taken for analysis at certain time points which included a total replacement of the PBS so as to allow the calculation of cumulative release. Experiments were performed in triplicate and average values were calculated.

### Cytotoxicity Analysis

Cells were plated in a 96 well plate at a density of 10,000 cells per well 24 hours prior to experimentation. A stock solution of the nanotubes was made up in media and sonicated briefly immediately before performing the serial dilutions (240 μg/mL down to 30 μg/mL). The media in each well was removed and replaced either with 100 μL the nanotubes at the variety of concentrations, or 100 μL of media alone for the negative control. After 24 hours, the cells were imaged using a light microscope and then washed two times with PBS. A 10% solution of PrestoBlue® in media was added to each well and incubated either for 1 hour (astrocytes) or 30 minutes (SHSY-5Y cells) until the color change from blue to pink could be observed in the control wells. The fluorescence was read with a plate reader at an excitation wavelength of 540 nm and an emission wavelength of 590 nm, and changes in fluorescence were normalized to control wells reported as 100% viable. In addition, CLSM was used to assess the uptake of doxorubicin loaded nanotubes in neuroblastoma cells at two different nanotube concentrations (30 μg/mL and 240 μg/mL). A 20× lens was used to image clusters of nanotubes within SHSY-5Y cells, via an overlay of FITC filter (doxorubicin) and brightfield images.

### Magnetic Focusing of Nanotubes

For these experiments the well plate set up was the same as described above except that the well plate was placed on top of a magnetic stir plate (placed within the cell culture incubator) for the duration of the incubation period (24 hours). Prior to the study, a 96 well plate containing small stir bars was placed at a specific point where the direction of nanotube movement could be predicted within the well. The stirring speed was set to a slow 100 rpm to assist the movement of the nanotubes. After the incubation period, the well plate was removed from the stir plate and observed by light microscopy.

## Additional Information

**How to cite this article**: Newland, B. *et al.* Magnetically Controllable Polymer Nanotubes from a Cyclized Crosslinker for Site-Specific Delivery of Doxorubicin. *Sci. Rep.* **5**, 17478; doi: 10.1038/srep17478 (2015).

## Supplementary Material

Supplementary Information
