# Supplementary material for: Magnetically Controllable Polymer Nanotubes from a Cyclized Crosslinker for Site-Specific Delivery of Doxorubicin
Source: Sci Rep. 2015 Dec 1;5:17478. doi: 10.1038/srep17478 (PMC4664922; doi:10.1038/srep17478)
Supplement: Supplementary Information [file srep17478-s1.doc]

Magnetically Controllable Polymer Nanotubes from a Cyclized Crosslinker for Site-Specific Delivery of Doxorubicin.

Ben Newland, Daniel Leupelt, Yu Zheng, Laurent S.V. Thomas, Carsten Werner, Martin Steinhart, Wenxin Wang.

**Supplementary Information**

**Homopolymerization of EGDMA by *in situ* DE-ATRP**

The monomer (EGDMA), initiator (EBriB), catalyst (CuCl_2_), and ligand (PMDETA) were added to two necked round bottom flask in the ratios shown in SI Table 1:

**SI Table 1** – Molar ratios of reagents with corresponding mass in grams.

|  | EBriB | EGDMA | CuCl_2_ | PMDETA | AA | Butanone |
| --- | --- | --- | --- | --- | --- | --- |
| Ratio (equiv) | 1 | 50 | 0.125 | 0.125 | 0.025 | - |
| N^o^ of Moles (mmol) | 2 | 100 | 0.25 | 0.25 | 0.05 | - |
| Mass (g) | 0.39 | 19.8 | 0.033 | 0.045 | 0.0088 | 50 mL |

A magnetic stir bar and 50ml of 2-butanone was added then argon was bubbled through the resulting solution for 30 mins to remove oxygen. 0.088 mL of an AA solution at 100 mg/ml (in deionised water) was added into the flask using a microliter syringe under positive pressure from argon. The flask was then immersed in an oil bath at 50 ^o^C and stirred at 800 rpm for the desired reaction time.

**SI Table 2 –** Molecular weight and polydispersity index (PDI) analysis of samples taken from the reaction vessel during the reaction process showing the low PDI of the knot polymer at 4 hours (1.48), but high PDI two hours later as traditional intermolecular branching occurs (6 hours).

| **Time (hr)** | **% Conversion** | **M_n_ (kDa)** | **M_w_ (kDa)** | **PDI (M_w_/M_n_)** |
| --- | --- | --- | --- | --- |
| 1 | 4.17 | 1.88 | 2.40 | 1.28 |
| 3 | 23.3 | 4.11 | 5.92 | 1.44 |
| 4 | 35.1 | 7.71 | 11.42 | 1.48 |
| 5 | 45.5 | 10.39 | 21.62 | 2.08 |
| 6 | 52.7 | 16.71 | 50.12 | 3.00 |


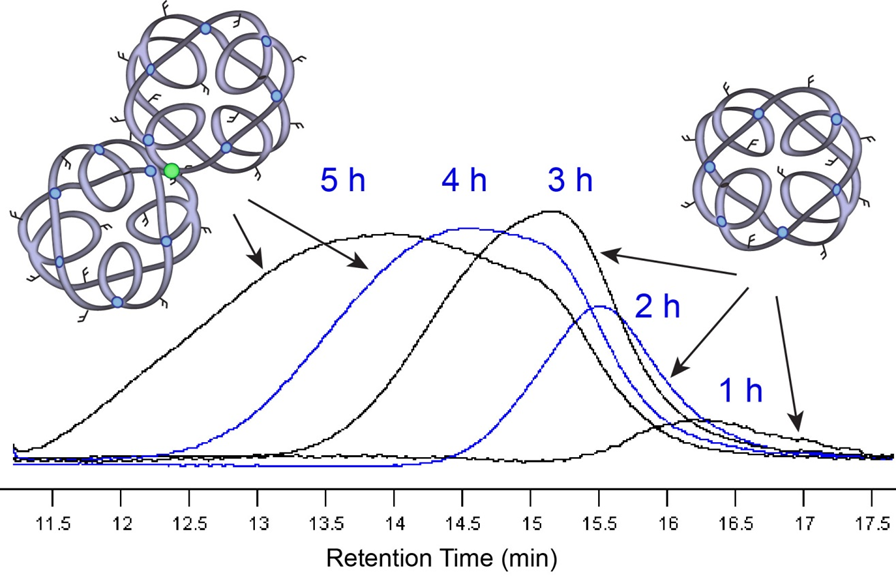


**SI Figure 1.** Gel permeation chromatography (GPC) analysis of the polymer synthesis during the reaction process. The narrow peaks of the knot polymer at the first phase of the reaction (up to 3 hours) gives way to broad peaks showing the polydisperse structures formed when these knots combine. **
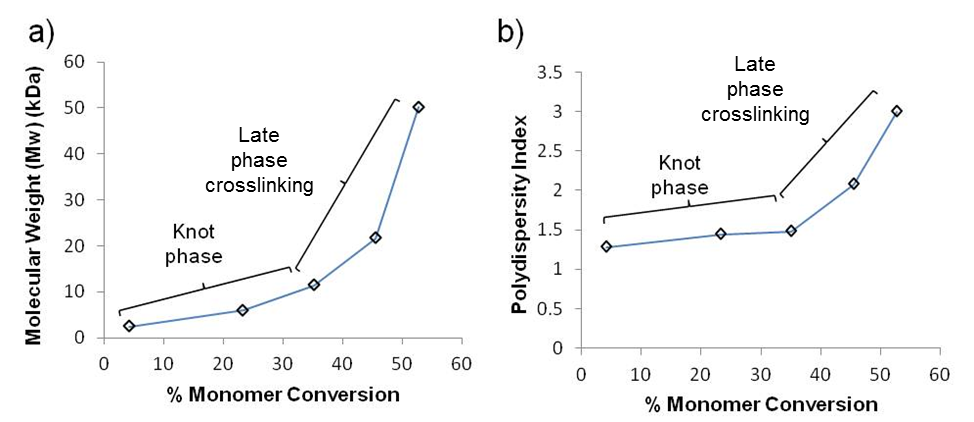
**

**SI Figure 2 –** Graphic representation of the change in Mw (a) or PDI (b) with increasing monomer conversion. The two distinct reaction phases are clearly shown, the first being the linear like growth of the knot structure, and the second occurring at higher conversion, when multiple knots combine by intermolecular reactions.


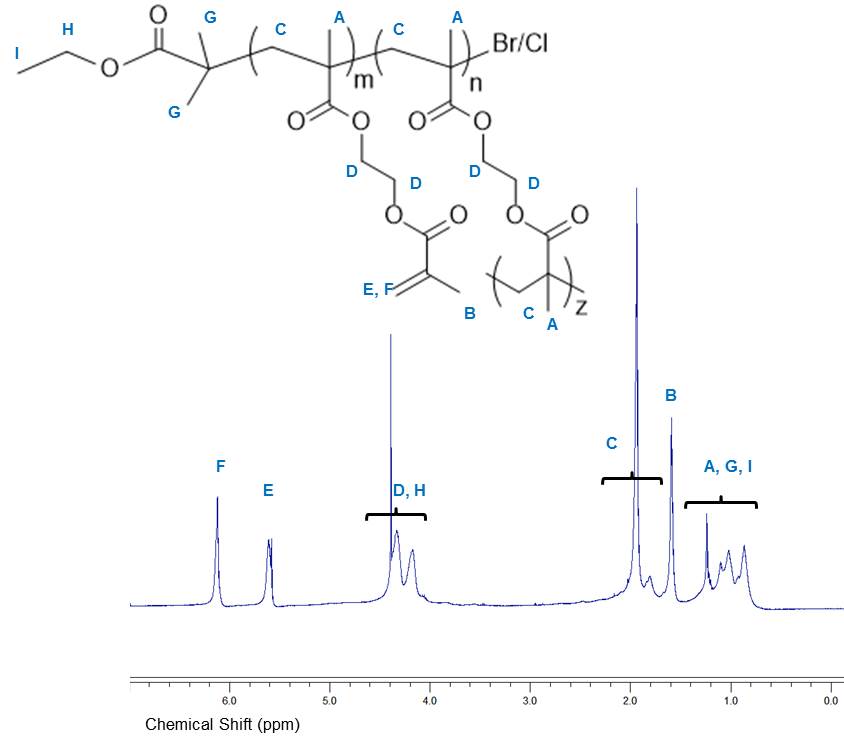


**SI Figure 3 –** ^1^H NMR data of the purified polymer with corresponding peak allocation. The two peaks near 6ppm clearly show the presence of vinyl groups within the polymer structure.

Percentage of the free vinyl containing EGDMA was calculated by dividing the vinyl content (integral of E), by the total EGDMA content (integral of D/4).


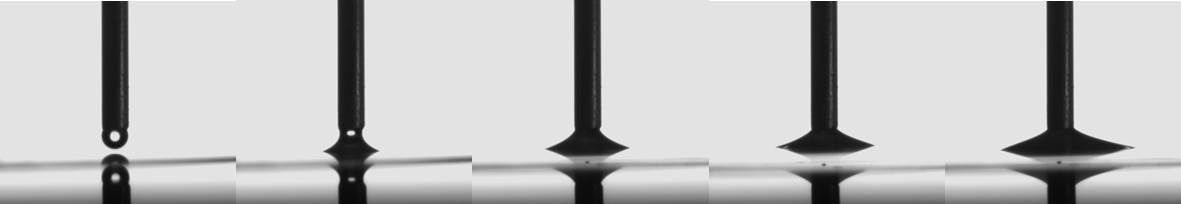


**SI Figure 4** – Analysis of the template wetting. Template wetting occurs as soon as the pre-polymer solution contacts the AAO template surface, as shown in the above sequence taken using DataPhysics OCA 20 apparatus (DataPhysics, Germany).


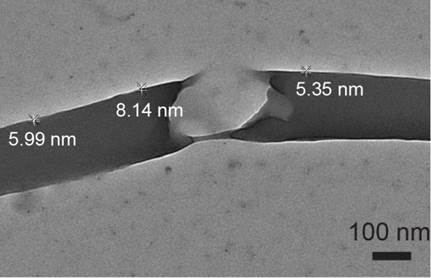


**SI Figure 5 –** TEM image of a rare wall defect in a polymer nanotube synthesized in the 200 nm pore template.


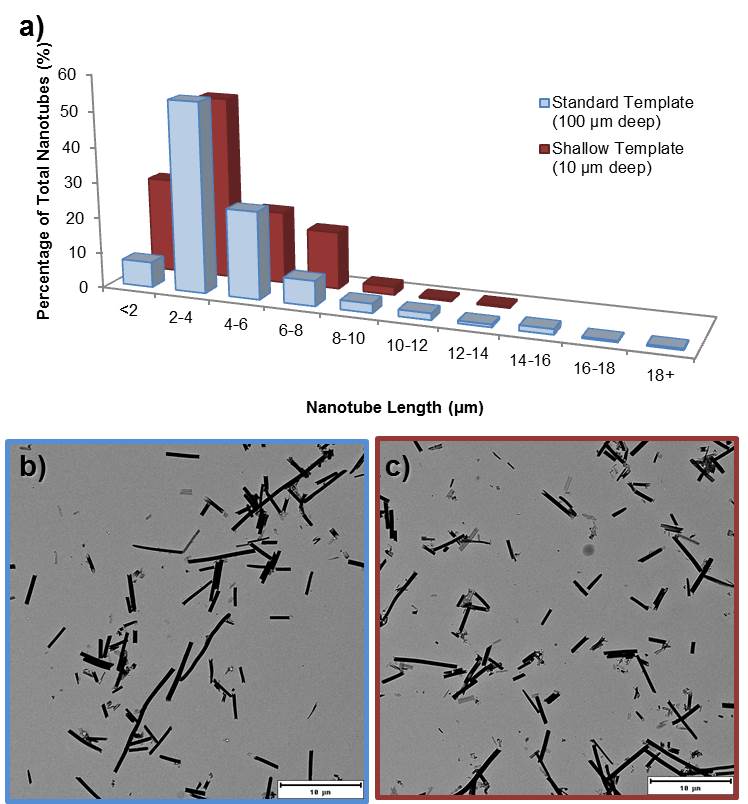


**SI Figure 6 – a)** The length distributions can be altered by the use of AAO templates with varying pore depth. TEM images of nanotubes from a standard template **(b)** where occasional long (˃10 µm) nanotubes can be seen, or a shallow template **(c)** where all nanotubes are under a maximum length as determined by the depth of the pores in the AAO template.


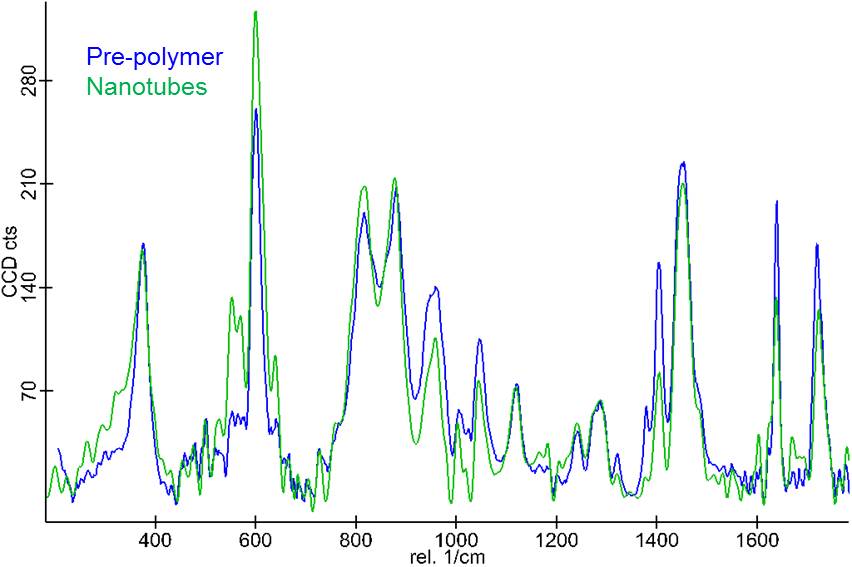


**SI Figure 7** – Raman spectroscopy analysis to assess the vinyl content before and after crosslinking the pre-polymer into the nanotube structure. There is a distinct, but small decrease in the peak height (1600 – 1800 cm-1) (quantified in the table below), showing the presence of vinyl groups in the nanotube structure.

**SI Table 3** – Analysis of the peak intensity and peak area for the data obtained by Raman spectroscopy, showing the decrease in vinyl content upon nanotube formation as shown by the reduction in the CH=/C=O ratio.

|  | **Intensity CH=** | **Intensity C=O** | **CH=/C=O** |
| --- | --- | --- | --- |
| Pre-polymer | 199.2 | 161.9 | 1.23 ± 0.05 |
| Nanotubes | 134.3 | 121.5 | 1.11 ± 0.05 |
|  |  |  |  |
|  | **Area CH=** | **Area C=O** | **CH=/C=O** |
| Pre-polymer | 2109 | 3825 | 0.55 ± 0.05 |
| Nanotubes | 1236 | 2892 | 0.43 ± 0.05 |


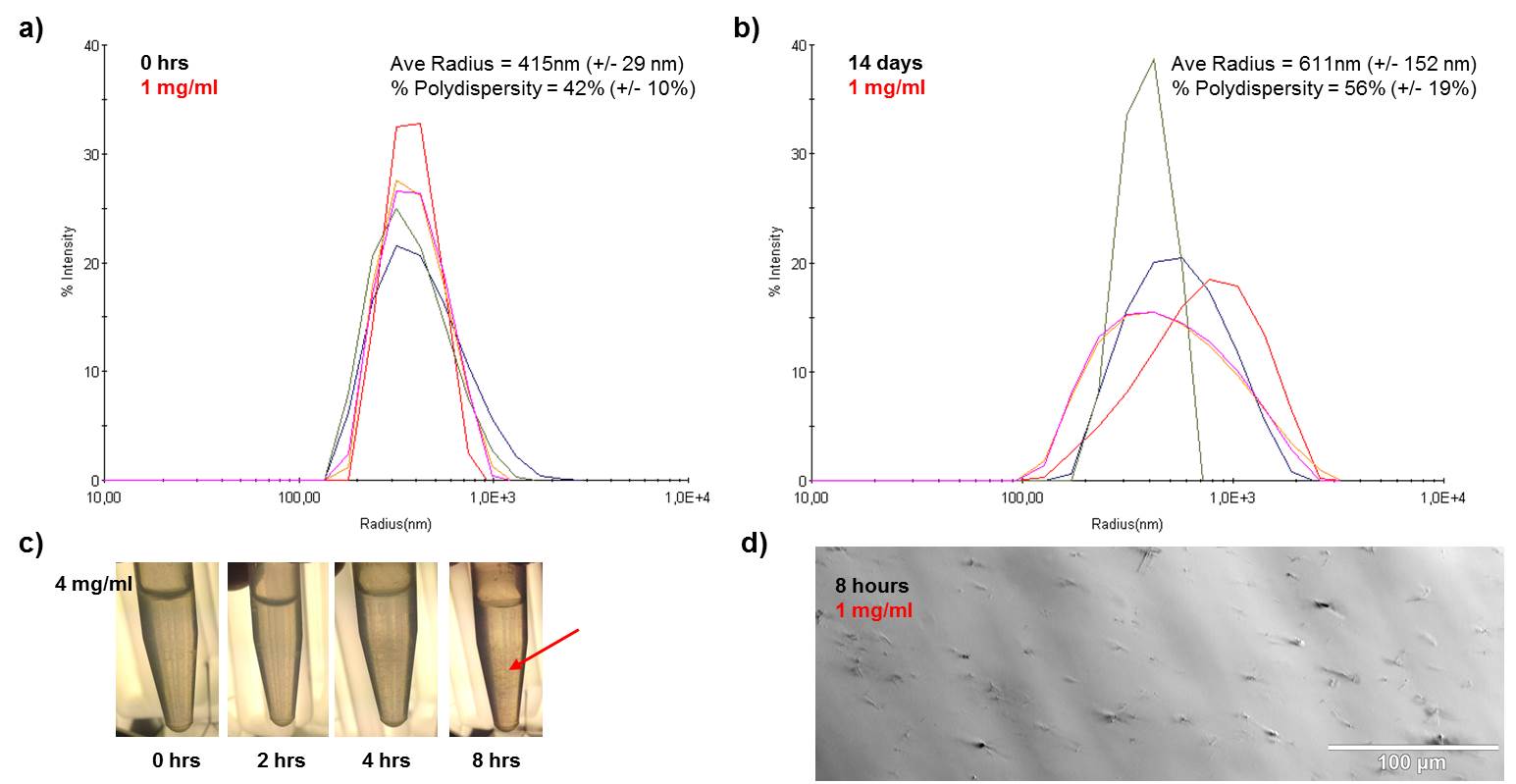


**SI Figure 8** – Dynamic light scattering analysis of the polymer nanotube dispersion at 1 mg/ml, after 0 hours **(a)** (freshly dispersed in PBS), and after 14 days **(b)**. Agglomeration can be observed by the increase in average radius and broadening of the peaks. For a visual representation a solution prepared at 4 mg/ml **(c)** was used so as to show aggregation after 6 hours and clustering of the nanotubes starting sedimentation at 8 hours. This high concentration was never used for cell experiments, and indeed even a 1 mg/ml in PBS could be observed dispersed through the solution above the well bottom **(d)** moving due to Brownian motion (hence blurred trails).


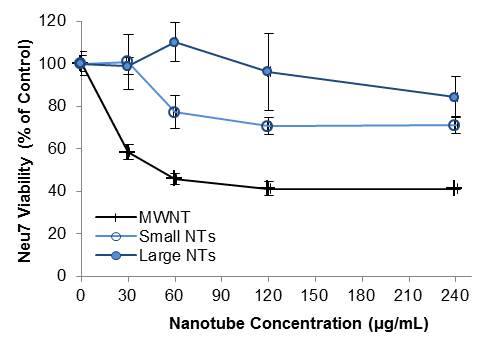


**SI Figure 9** – Analysis of the viability of Neu7 immortalized astrocytes after 24 hours incubation with varying concentrations of polymer nanotubes from the 200 nm AAO template (Small) and from the 400 nm template (Large). At all concentrations analyzed the polymer nanotubes were less toxic than the MWNT control group.


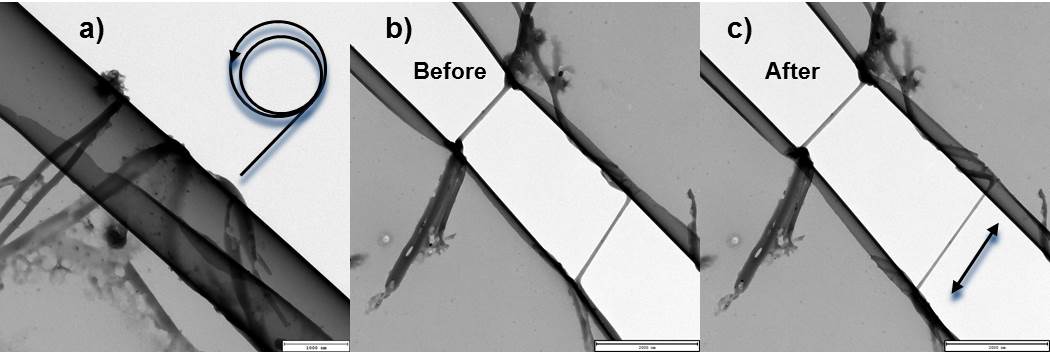


**SI Figure 10 –** TEM images showing two examples of the flexible nature of the polymer nanotubes. Where defects in the carbon membrane of the sample holding grid occur, nanotubes can be seen to “wrap up” **(a)** as the electron beam intensity causes the curling or the ripped carbon film. Not only are they flexible, but also can stretch as shown by the transition in length from image **(b)** to image **(c)**. Two nanotubes span the rip in the carbon film and as the electron beam intensity is increased the rip widens and the nanotubes stretch and become thinner. Scale bars are 1000, 2000 and 2000 nm respectively.


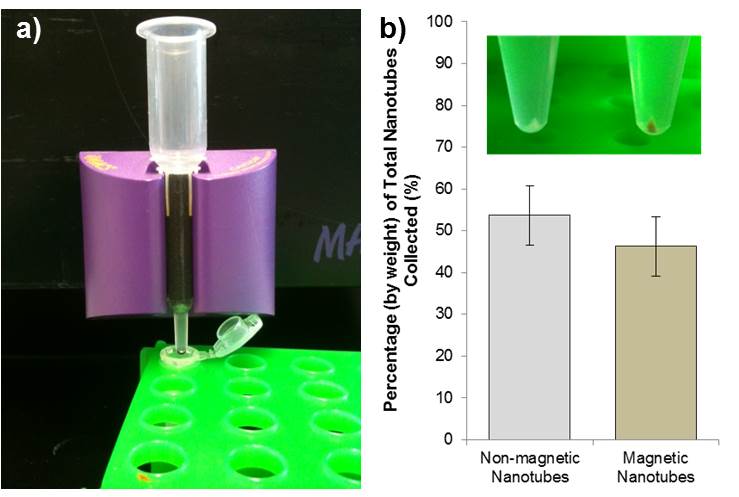


**SI Figure 11 –** Photographs, taken by the authors using a Nikon D90 camera, of the MACS miltenyi biotec equipment set up used to separate magnetic nanotubes from non-magnetic nanotubes **(a)** and subsequent pellets (**b** insert) showing non-magnetic nanotubes as a white pellet (left) and magnetic nanotubes as a brown pellet (right). Non-magnetic nanotubes pass through the filter to be collected in the first Eppendorf, whilst the magnetic nanotubes are retained in the filter until flushed (in the absence of the purple magnet) into a second Eppendorf. Pellet weights were compared for 6 different preparations and the average percentage of the total weight for each fraction was calculated **(b)**.

**SI Table 4** – Doxorubicin loading data from the analysis of three samples, showing that 38 ng of doxorubicin is loaded to 1 µg of polymer nanotubes, when a loading solution at 80 µg/ml of doxorubicin is used.

| Loading Data | Average | Standard  Deviation |
| --- | --- | --- |
| Amount of doxorubicin in NTs (µg) | 10.09 | 0.82 |
| % of loading solution in NTs (%) | 63.10 | 5.14 |
| Doxorubicin per NTs (ng/µg) | 38.39 | 3.12 |


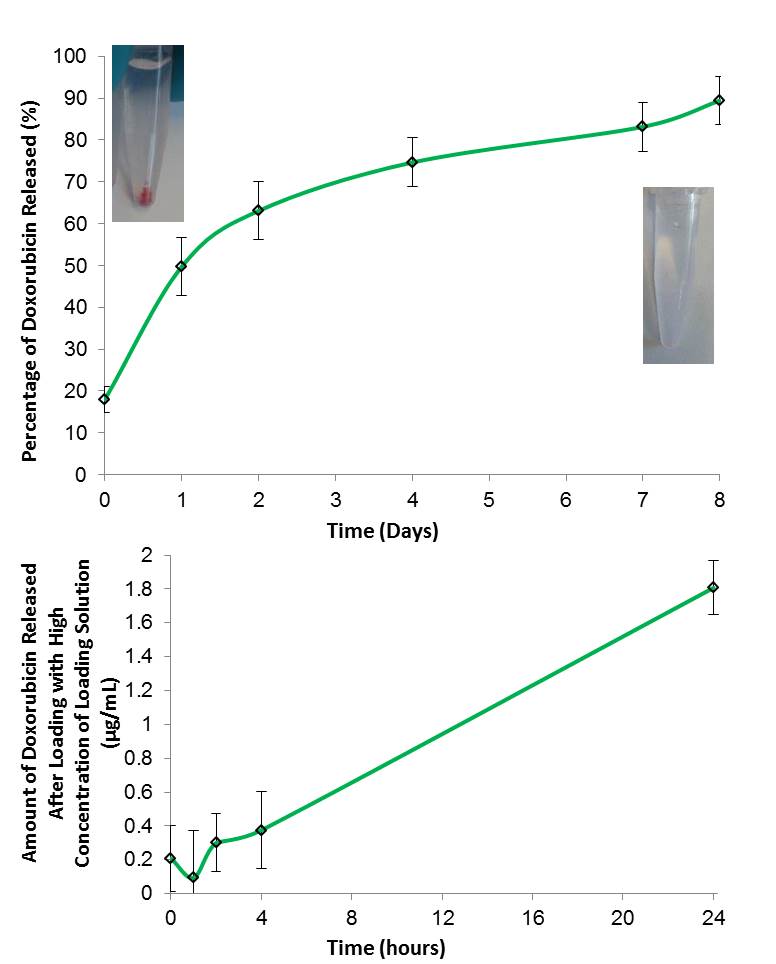


**SI Figure 12** – Analysis of the release rate of doxorubicin from nanotubes that had been loaded in a loading solution of 90 µg/mL (See **SI Table 4**) into PBS over a period of eight days at 37^o^C (top panel). The graph shows that doxorubicin is released from the nanotubes over time, leaving behind an almost clear pellet of empty nanotubes (insert images of pellet just as the experiment starts (left) and after eight days (right). For cytotoxicity experiments a high concentration of loading solution was used (1.5 mg/mL) and the lower panel shows the release rate of doxorubicin from nanotubes loaded with this highly concentrated loading solution as would be experienced by the cells during the cytotoxicity experiments (n=3, error bars represent +/- standard deviation).


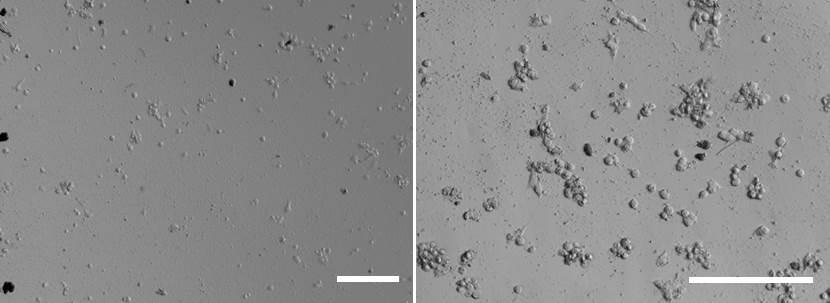


**SI Figure 13 –** Low magnification (left) and higher magnification (right) light microscopy images of doxorubicin loaded non-magnetic nanotubes after 24 hours incubation with SHSY-5Y cells, showing an even scattering of the nanotubes across the well surface (scale bars = 200 µm).


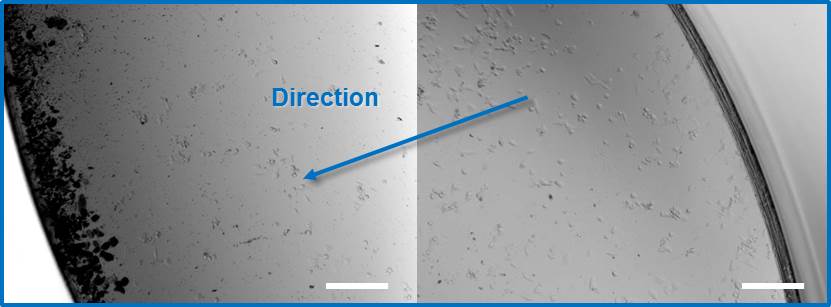


**SI Figure 14 –** Further light microscope images of the magnetic focusing of the magnetic nanotubes within the same well (of a 96 well-plate) which contains SHSY-5Y neuroblastoma. The left hand image shows the left side of the well plate (slightly below the centerline) which has many nanotubes and large aggregations at the well wall. In contrast, the right hand image shows the right side of the same well (slightly above the centerline) where no nanotubes can be observed. The approximate direction of the nanotube movement has been added for clarity (scale bars = 200 µm).


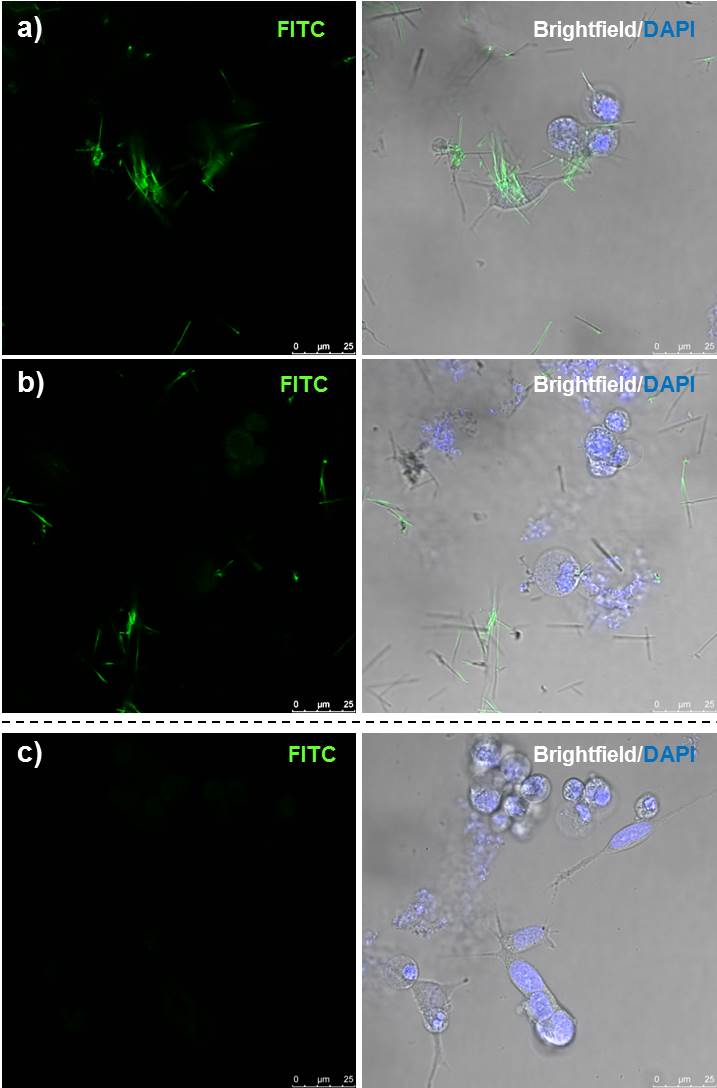


**SI Figure 15 –** Further images of SHSY-5Y cells incubated for 24 hours with non-labelled (i.e. not intrinsically fluorescent) but doxorubicin loaded nanotubes (doxorubicin fluorescence observed at 488 nm) using DAPI as a nuclear stain. Some cells still appear unaffected (panel **a**), but the majority of cells are disintegrated (panel **b**), rounded or large clear patches devoid of cells can be observed. A doxorubicin control of 2 µg/mL with no nanotubes also showed dead/dying cells (panel **c**). Fluorescence in the cells due to the up-taken doxorubicin could not be observed for any of the samples indicating that the level needed for cell death is below the fluorescence threshold used.


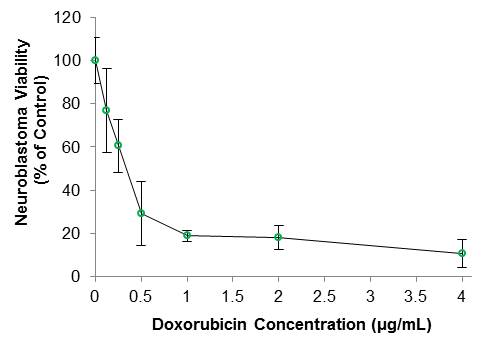


**SI Figure 16 –** Analysis of the effect of free doxorubicin on SHSY-5Y cell viability after incubation for 24 hours to allow comparison with toxicity data obtained for the doxorubicin released from the nanotubes (n=4, error bars represent +/- standard deviation).
